# Clinical applications and limitations of current ovarian stem cell research: a review

**DOI:** 10.1186/1743-1050-3-6

**Published:** 2006-07-27

**Authors:** Karla J Hutt, David F Albertini

**Affiliations:** 1 Center for Reproductive Sciences, Department of Molecular and Integrative Sciences, University of Kansas Medical Center, Kansas City, KS 66160, USA; 2 Marine Biological Laboratory, Woods Hole 02543, MA, USA

## Abstract

The publication of a report in Nature in 2004 by the Tilly group suggesting that mouse ovaries are capable of generating oocytes de novo post-natally, has sparked interest in a problem long thought to have been resolved from classical studies in a variety of mammalian species. Within a nearly two year time period, laboratories around the world have taken up the challenge to dogma raised by this initial report, either to test this concept in an experimental basic science setting or give direction to clinical applications that could result, were the original premises of this work in the mouse valid for extrapolation to humans. This review provides a status report for this promising area of research, (1) to summarize recent findings in the literature with respect to the validity of the original hypothesis proffered by the Tilly group, and, (2) to gauge the potential utility of ovarian stem cells as a treatment for certain forms of human infertility.

## The science then and now

The background for this debate derives primarily from two papers (Table 1). Taken together, these studies [1,2] reach the conclusion that an oocyte replenishing mechanism must exist in the mouse ovary after birth to explain variations in follicle number detected after experimental manipulations, and during ovarian development or the estrous cycle. Besides drawing upon follicle counting methodology in a variety of conditions (animal age, cycle status, recovery from ovotoxicity), expression marker data were published prompting models of either an ovarian epithelium-based progenitor cell for germline derivation of new follicles [1] or a bone marrow derived stem cell competent to hone into the ovary and generate new follicles concomitant with meiotic entry for such precursor populations [2]. While the intriguing demonstration of stem/germline markers in human blood cells remains uncontested, by and large all recent efforts to study this problem have maintained focus on the murine model and are summarized below.

**Table 1:** Recent articles in the area of germline stem cells

**Authors**	**Year**	**Main findings**
Johnson et al.	2004	Germline stem cells are located within the ovarian epithelium and supply the adult mouse ovary with new oocytes.
Johnson et al.	2005	Germline stem cells are present in the circulation and supply the adult mouse ovary with new oocytes.
Byskov et al.	2005	Failed to find any BrdU positive oogonia within the ovarian epithelium and provided evidence to suggest that an overestimation of atretic follicle number contributed to Johnson et al.'s conclusions.
Oktay et al.,	2005	Reported spontaneous pregnancy in presumably sterile patient following chemotherapy, hematopoeitic stem cell transplantation and ovarian tissue transplantation.
Bristol-Gould et al.	2006	Showed that follicle numbers gradually decline in the mouse ovary with age and used mathematical modeling to determine that de novo follicle production is not required to support fertility.
Eggan et al.	2006	Used parabiotic mice to show that ovulated oocytes do not come from circulating stem cells.
Kerr et al.	2006	Showed that follicle numbers remain constant in mouse ovaries from puberty to early mid-life, suggesting possible follicular renewal.

### Follicle counting

Three studies have appeared that address the question of follicle dynamics and the likelihood of post-natal de novo follicle production (Table 1). Of these, the recent article by Kerr et al. [3] lends indirect support to the concept of ongoing oocyte regeneration within the ovary. By using unbiased stereological techniques to compare follicle numbers in ovaries from neonatal and adult mice belonging to the same strain studied by Johnson et al. [1,2], maintenance of primordial follicle numbers during early post-natal life through to middle age is demonstrated, rather than progressive follicle loss over time reported in previous studies. Although the persistence of follicle numbers points to a mechanism for sustaining the oocyte pool in mouse ovaries over their reproductive lifespan, these authors found no histological evidence for the existence of ovarian germline stem cells. Drawing on stereological measurements then raises questions about the validity of alternative follicle counting methods and the definition of healthy versus atretic follicles as assayed by either approach.

The original hypothesis for oocyte and follicular renewal takes its roots from a perceived conflict between the actual numbers of healthy and atretic follicles present in the ovary, compared to estimations made for the rate of follicle loss [1]. In addition to providing a number of reasons for why the assumptions and mathematical equations used in this study were inherently flawed, Byskov et al. [4] performed their own experiments to examine the characteristics and rate of follicle atresia and in doing so arrive at alternative explanations for the Tilly groups findings. Byskov et al. [4] report evidence to indicate that atretic follicles are actually cleared from the ovary more slowly than originally calculated, and that the pool of atretic follicles found on day 30 by Johnson et al. [1] likely includes growing follicles that had degenerated many days earlier. Thus, the claim that in the absence of continuous follicular regeneration the ovarian pool of oocytes would be prematurely depleted may in fact be partially attributed to the authors' miscalculation of the rate of atretic follicle clearance.

It seems clear that a thorough understanding of follicular dynamics, defined by the nature of both follicular progression and follicular loss, is needed before any conclusions about the existence of germline stem cells can be inferred from static studies of follicle numbers at selective time points. With this in mind, Bristol-Gould et al. [5] used sophisticated mathematical models, capable of describing follicular dynamics within the ovary, to determine if replenishment of the initial primordial follicle pool is required to support fertility throughout reproductive life. First, they counted follicles in ovaries of mice ranging from day 6 through to 12 months. They then applied two models to their experimental data: the fixed pool model, which assumes the pool of primordial follicles established in the ovary before birth is non-renewable; and the stem cell model, which predicts that de novo follicle production supplements the initial follicle pool. Despite manipulating the latter to account for different rates of oocyte production, Bristol-Gould et al. [5] found that only the fixed pool model accurately reflected the observed gradual depletion of follicle numbers over time. Their results show that the initial store of primordial follicles is indeed sufficient to supply the mouse with all the experimentally observed oocytes, and that supplementation of this original pool, in terms of number alone, is not necessary.

With respect to stem cell behaviors, two studies are relevant (Table 1). First, Byskov and colleagues [4] have repeated the BrdU detection paradigm (using identical mouse strains as the Tilly group) and found no evidence for nascent DNA synthesis over a wide range of animal ages. That the ovarian epithelium is a site of new germ cells/follicle formation is unlikely in view of this new and strikingly confirmatory data as it argues strongly for the lack of a typical regenerative mechanism in the mouse ovary. The paucity of studies on marker expression such as those used by Johnson et al. [1] does however mean that if such events are rare enough to escape detection by DNA precursor loading, then these and other reagents should be deployed to satisfactorily resolve this point. Similarly, images purporting to demonstrate germ cells in the epithelium, historically construed as being involved in oocyte loss [6], remain enigmatic and will require reassessment.

The second study from Eggan and colleagues [7] addresses the question of bone marrow precursors to new oocytes. Here, elegant and again historically useful approaches employing both parabiosis between donor and host animals and the bone marrow transplant procedures used by Johnson et al. [2] form the basis for asking a simple and oft sought after question (see commentaries by Telfer et al. [8,9]): Can genetically marked bone marrow or blood borne stem cells give rise to ovulated oocytes? While both parabiotic and bone marrow transplant animals continued to respond to gonadotropins and supported induced ovulation with high efficiency, these investigators were unable to document ovulation of oocytes that would have been derived from the appropriate donor tissues. More telling perhaps was the finding that after chemical induced oocyte ablation, sufficient stores of unaffected oocytes were detected after induced ovulation further suggesting, as have others, that the methods used to achieve total oocyte reduction were incomplete at best. This point will be further discussed with respect to recent reports in humans who have re-established pregnancies following chemotherapy treatments.

## Of mice and women

Summarily then, the evidence taken from recent studies argues against the existence of an ovarian stem cell mechanism that could support follicle replenishment... at least in mice. Questions of the appropriateness of murine models to study human reproductive physiology notwithstanding, we would like to return to the topic of stem cells and humans as this has received much attention in the popular press, apparently at the expense of "hard science" from the medical community. In our estimation, the most intriguing studies are those of Oktay and colleagues who have been pioneers in the development ovarian restoration protocols to assist young women who have undergone sterility-inducing cancer treatments. Heterologous bone marrow transplant has been used for years in the management of such patients and several reports of restored fertility have appeared in the literature [10-12]. More recently, Oktay [13,14] has reported a cancer patient who, after experiencing more than two years of menopause as a consequence of receiving sterilizing chemotherapy prior to hematopoeitic stem cell transplantation, conceived twice immediately following transplantation of her own cryopreserved ovarian tissue. The origin of the pregnancies are unknown, but were likely due to the spontaneous recovery of her existing ovary. The potential contribution of circulating germline stem cells to these pregnancies remains unexplored. The case for a blood borne/bone marrow stem cell capable of replenishing the human ovary require serious consideration in view of reports of this kind and all efforts to translate cases of this nature into a treatment strategy should be encouraged and pursued. Surely, were this the case, then appropriate genetic evidence can and should be brought to bear on the thousands of patients who have conceived following chemotherapy and these results are ones all in this field are anticipating.

## Conclusion

Evidence from a number of laboratories collectively raise concerns over the validity of the ovarian stem cell mechanism for follicle replenishment as originally proposed by Johnson et al. [1,2]. However, recent reports on fertility restoration in humans are promising and warrant further study as hopes for novel fertility techniques are legitimized and the prospect that germline regeneration, while unlikely to occur in mice, may have some basis in primates.

## Competing interests

The author(s) declare that they have no competing interests.

## Authors' contributions

Karla Hutt and David Albertini contributed equally to the writing of this manuscript.
